# Impact of preadmission treatment with calcium channel blockers or beta blockers on short-term mortality after stroke: a nationwide cohort study

**DOI:** 10.1186/s12883-015-0279-3

**Published:** 2015-03-07

**Authors:** Jens Sundbøll, Morten Schmidt, Erzsébet Horváth-Puhó, Christian F Christiansen, Lars Pedersen, Hans Erik Bøtker, Henrik T Sørensen

**Affiliations:** Department of Clinical Epidemiology, Aarhus University Hospital, Olof Palmes Allé 43-45, Aarhus N, DK-8200 Denmark; Department of Cardiology, Aarhus University Hospital, Skejby, Brendstrupgårdsvej 100, Aarhus N, DK-8200 Denmark

**Keywords:** Beta-blocker, Calcium channel blocker, Stroke, Ischemic stroke, Hemorrhagic stroke, Mortality

## Abstract

**Background:**

The prognostic impact of preadmission use of calcium channel blockers (CCBs) and beta blockers (BBs) on stroke mortality remains unclear. We aimed to examine whether preadmission use of CCBs or BBs was associated with improved short-term mortality following ischemic stroke, intracerebral hemorrhage (ICH), or subarachnoid hemorrhage (SAH).

**Methods:**

We conducted a nationwide population-based cohort study using Danish medical registries. We identified all patients with a first-time inpatient diagnosis of stroke between 2004 and 2012 and their comorbidities. We defined CCB/BB use as current use, former use, or non-use. Current use was further classified as new or long-term use. We used Cox regression modeling to compute 30-day mortality rate ratios (MRRs) with 95% confidence intervals (CIs), controlling for potential confounders.

**Results:**

We identified 100,043 patients with a first-time stroke. Of these, 83,736 (83.7%) patients had ischemic stroke, 11,779 (11.8%) had ICH, and 4,528 (4.5%) had SAH. Comparing current users of CCBs or BBs with non-users, we found no association with mortality for ischemic stroke [adjusted 30-day MRR = 0.99 (95% CI: 0.94-1.05) for CCBs and 1.01 (95% CI: 0.96-1.07) for BBs], ICH [adjusted 30-day MRR = 1.05 (95% CI: 0.95-1.16) for CCBs and 0.95 (95% CI: 0.87-1.04) for BBs], or SAH [adjusted 30-day MRR = 1.05 (95% CI: 0.85-1.29) for CCBs and 0.89 (95% CI: 0.72-1.11) for BBs]. Former use of CCBs or BBs was not associated with mortality.

**Conclusions:**

Preadmission use of CCBs or BBs was not associated with 30-day mortality following ischemic stroke, ICH, or SAH.

**Electronic supplementary material:**

The online version of this article (doi:10.1186/s12883-015-0279-3) contains supplementary material, which is available to authorized users.

## Background

Stroke is one of the most disabling neurological diseases [1]. It is expected to remain the second leading cause of death worldwide, exceeded only by myocardial infarction [1-3]. From 1999 to 2009, the annual incidence of first-time stroke was approximately 600,000 cases in the United States [4]. Thirty-day mortality is approximately 11% for ischemic stroke [5], 34% for intracerebral hemorrhage (ICH) [5], and 29% for subarachnoid hemorrhage (SAH) [6].

Calcium channel blockers (CCBs) and beta blockers (BBs) are commonly used antihypertensive agents [7]. It has previously been established that elevated preadmission blood pressure is associated with markedly increased stroke mortality [8]. Because the association between blood pressure at hospitalization and mortality is U-shaped in stroke [9], and both ischemic and hemorrhagic strokes are associated with transiently elevated blood pressure in the acute phase [10], we hypothesized that preadmission use of CCBs or BBs could reduce short-term mortality through improved blood pressure control.

It is well established that CCBs and BBs reduce stroke risk in persons with hypertension [11], but it remains unknown whether these agents have any effect on mortality following stroke. In a recent study, it was established that angiotensin-converting enzyme inhibitors and angiotensin receptor blockers were associated with reduced 30-day mortality following ischemic stroke but not haemorrhagic stroke [12]. To investigate whether this protective effect extended to other antihypertensive agents, we examined whether use of CCBs or BBs at time of stroke was associated with reduced short-term mortality.

## Methods

### Setting

We conducted this nationwide population-based cohort study in Denmark. During the study period (1 July 2004 to 31 December 2012), the total underlying population at risk was 6,379,918 inhabitants. The study period commenced 6 months after establishment of the Danish National Database of Reimbursed Prescriptions (DNDRP) on 1 January 2004, to ensure availability of at least 6 months of preadmission prescription history for all participants [13]. The Danish National Health Service provides universal tax-supported health care, guaranteeing unfettered access to general practitioners and hospitals and partial reimbursement for prescribed medications, including CCBs and BBs [13]. We linked medical registries using the unique central personal registry number assigned to every Danish citizen at birth and to residents upon immigration [14].

### Patients with stroke

In Denmark, care for patients with stroke and other medical emergencies is provided by public hospitals [15]. We used the Danish National Registry of Patients (DNRP), covering all Danish hospitals [16], to identify all patients with a first-time inpatient hospitalization for stroke during the study period. Because more than two-thirds of all unspecified strokes (International Classification of Diseases (ICD-10) code: I64) are known to be ischemic strokes (ICD-10 code: I63) [17], we classified unspecified strokes (32% of all stroke diagnoses [12]) as ischemic strokes. The DNRP contains data on admission and discharge dates and discharge diagnoses from all non-psychiatric hospitals since 1977 and on emergency room and outpatient clinic visits since 1995 [16]. Each hospital discharge is assigned one primary diagnosis and up to 19 secondary diagnoses classified according to the *International Classification of Diseases*, 8th revision (ICD-8) until the end of 1993 and 10th revision (ICD-10) thereafter [16]. We identified patients using both primary and secondary diagnoses for ischemic stroke, ICH, and SAH.

### CCB and BB use

We used the DNDRP to identify all prescriptions redeemed for CCBs and BBs by study participants [13]. Pharmacies in Denmark are equipped with electronic accounting systems, primarily used to secure reimbursement from the National Health Service. For each redeemed prescription, the patient’s central personal registry number, the amount and type of drug prescribed according to the Anatomical Therapeutic Chemical (ATC) classification system, and the date the drug was dispensed are transferred electronically from the pharmacies to the DNDRP [13].

We defined current users as patients whose last prescription redemption for CCBs or BBs was <90 days before hospital admission for stroke. We chose an exposure window of 90 days to identify most current users, as prescriptions of CCBs/BBs are seldom provided for more than 90 days at a time in Denmark. As the drugs’ effects and potential side effects may be less pronounced in long-term users [18], this could lead to underestimation of the association with mortality [18]. Consequently, current users were further categorized into new users, who redeemed their first-ever CCB or BB prescription within 90 days before their admission date, and long-term users, who redeemed their first-ever prescription more than 90 days before their admission date. We defined former users as patients whose last prescription redemption was between 90 and 180 days before admission, and non-users as patients with no prescription redemption within 180 days before admission.

### Mortality

We used the Danish Civil Registration System (CRS) to obtain information on all-cause mortality [14]. The CRS has recorded all changes in vital status and migration for the entire Danish population since 1968, with daily electronic updates [14].

### Patient characteristics

The complete inpatient and outpatient medical history available in the DNRP [16] provided information on known prognostic factors (myocardial infarction, atrial fibrillation or flutter, intermittent arterial claudication, diabetes, and dementia) [19] and other potential confounders (Table 1).

**Table 1:** **Characteristics of stroke patients by preadmission use of calcium channel blockers or beta blockers** ^**a**^

	**Ischemic stroke (n = 83736)**				**Intracerebral hemorrhage (n = 11779)**				**Subarachnoid hemorrhage (n = 4528)**			
	**Calcium channel blocker**		**Beta blocker**		**Calcium channel blocker**		**Beta blocker**		**Calcium channel blocker**		**Beta blocker**	
	**Current use n = 13170 (100%)**	**Non-use n = 67571 (100%)**	**Current use n = 17189 (100%)**	**Non-use n = 63402 (100%)**	**Current use n = 1163 (100%)**	**Non-use n = 10277 (100%)**	**Current use n = 1913 (100%)**	**Non-use n = 9494 (100%)**	**Current use n = 365 (100%)**	**Non-use n = 4051 (100%)**	**Current use n = 413 (100%)**	**Non-use n = 4023 (100%)**
**Male**	47.7	52.2	46.7	52.7	51.8	51.8	51.4	51.7	44.7	42.1	46.2	42.1
**Age, years**												
<60	9.7	20.7	9.2	21.6	12.5	26.0	11.5	27.3	24.1	57.1	24.2	57.5
60-69	19.7	21.9	19.5	22.1	21.0	20.9	20.0	21.0	23.8	20.9	26.6	20.6
70-79	30.8	26.2	30.7	25.9	31.8	25.2	33.3	24.0	25.5	12.6	24.2	12.4
≥80	39.8	31.2	40.5	30.5	34.7	27.9	35.2	27.7	26.6	9.4	24.9	9.4
**Comorbidities**												
Cardiovascular diseases												
Congestive heart failure	10.3	7.9	18.6	5.3	9.8	5.6	16.6	3.8	5.2	2.8	15.7	1.6
Myocardial infarction	11.5	8.2	21.6	4.8	11.6	5.3	17.8	3.3	7.1	2.7	18.2	1.4
Angina pectoris	24.7	13.4	33.1	10.0	25.1	10.1	30.4	7.5	21.1	5.7	32.4	4.1
Atrial fibrillation or flutter	19.5	12.1	30.3	8.5	23.6	11.8	38.2	7.5	15.1	4.3	24.7	2.9
Heart valve disease	6.4	4.0	8.6	3.2	6.7	3.7	11.0	2.6	4.7	1.6	6.8	1.3
Intermittent claudication	5.1	2.6	4.9	2.5	4.0	1.5	3.6	1.4	2.7	1.0	2.7	1.0
Venous thromboembolism	4.0	3.7	4.2	3.6	4.6	3.3	4.7	3.2	2.5	2.0	3.9	1.8
Hypertension	50.5	19.3	47.4	18.4	55.3	18.8	52.1	16.7	47.9	10.5	46.0	10.3
Other diseases												
Obesity	5.3	3.6	5.4	3.5	7.4	2.7	6.2	2.5	5.2	2.9	5.8	2.7
Diabetes mellitus	20.0	11.7	19.4	11.5	19.1	8.2	18.3	7.5	14.2	4.8	13.3	4.6
Chronic kidney disease	4.8	2.1	5.0	1.8	8.5	2.0	7.0	1.8	6.0	1.0	5.8	1.1
Chronic pulmonary disease	24.4	19.9	21.5	20.5	23.7	18.3	20.6	18.5	28.8	16.8	24.2	17.3
Alcoholism-related disease	4.9	6.8	5.2	6.8	5.8	8.4	6.2	8.4	5.5	7.6	10.4	7.1
Dementia	3.7	3.3	3.2	3.4	4.6	3.9	3.6	4.0	2.2	1.2	2.2	1.3
Cancer	16.2	14.0	16.2	13.9	18.7	15.3	18.6	15.0	16.4	8.0	18.2	7.8
**Concurrent medications**												
Cardiovascular drugs												
ACE-Is/ARBs	45.8	21.9	44.9	20.6	43.5	17.3	44.4	15.2	41.1	12.0	45.3	11.4
Beta blockers	31.8	18.0	-	-	28.4	14.7	-	-	23.6	7.5	-	-
Calcium channel blockers	-	-	24.4	13.3	-	-	17.3	8.3	-	-	20.8	6.6
Diuretics	44.4	24.2	48.8	21.8	40.3	19.0	43.7	16.5	32.6	11.0	39.5	9.9
Nitrates	4.3	1.4	5.2	1.0	3.9	1.0	3.9	0.6	2.5	0.4	3.9	0.1
Statins	28.1	13.7	31.9	11.7	30.8	11.9	31.4	10.3	25.8	8.5	33.9	7.5
Aspirin	38.9	21.1	45.8	18.2	37.7	18.9	42.1	16.5	29.6	8.2	35.1	7.2
Clopidogrel	3.1	1.6	4.7	1.1	3.1	1.3	4.6	0.9	1.9	0.9	4.8	0.5
Vitamin K antagonists	7.7	4.1	10.9	2.9	19.1	8.9	29.0	5.9	8.5	2.9	16.2	1.9
Other drugs												
Systemic glucocorticoids	5.8	4.6	5.2	4.7	4.8	3.8	5.2	3.7	6.3	2.4	3.9	2.6
SSRIs	10.6	8.7	10.1	8.7	11.2	9.5	12.7	9.2	9.3	6.9	11.9	6.5
Bisphosphonates	4.0	3.3	4.0	3.3	4.1	3.7	4.5	3.6	5.2	2.3	3.9	2.2
NSAIDs	12.1	10.7	11.2	10.9	11.4	10.2	9.4	10.6	15.1	10.4	11.4	10.8

^a^n = 100,043. Table values are given as %. Data for former users are not shown (n = 2995/3145 among ischemic stroke patients, 339/372 among intracerebral hemorrhage patients, and 112/92 among subarachnoid hemorrhage patients for calcium channel blockers/beta blockers, respectively). ACE-Is/ARBs indicates angiotensin converting enzyme inhibitors/angiotensin receptor blockers; SSRIs, selective serotonin reuptake inhibitors; NSAIDs, nonsteroidal anti-inflammatory drugs.

To increase the sensitivity of diagnoses of diabetes, COPD, and alcoholism-related diseases, we searched the DNDRP for any previous prescriptions for anti-diabetic medications, respiratory medications, and alcohol deterrents. Relevant ICD and ATC codes are provided in Additional file 1: Table S1.

### Comedication use

We obtained information from the DNDRP on concurrent use (last prescription redemption within 90 days before admission) of other drugs that may potentially affect stroke prognosis [13]. Relevant ATC codes are provided in Additional file 1: Table S1.

### Statistical analysis

We characterized the stroke cohort according to sex, age group (<60, 60–69, 70–79, ≥80 years), individual comorbidities, and comedication use. We followed all patients from hospital admission date until death, emigration, or 30 days of follow up, whichever came first.

We used a Cox proportional-hazards regression analysis to compute the hazard ratio as a measure of the mortality rate ratio (MRR) within 30 days of hospital admission for current, new, long-term, and former use compared with non-use. The analysis was adjusted for sex, age groups, and the individual comorbidities and comedications listed in Table 1. The proportional hazard assumption was assessed using log–log plots and found valid. We repeated the analysis stratifying by sex, age group, and presence/absence of myocardial infarction, congestive heart failure, atrial fibrillation or flutter, angina pectoris, hypertension, diabetes, and chronic kidney disease.

We performed four sensitivity analyses. First, to increase the positive predictive value (PPV) of the stroke diagnosis, we restricted the analyses to patients who had a computed tomography (CT) or magnetic resonance imaging (MRI) scan registered in the DNRP during the stroke admission. Second, to examine the sensitivity of the estimates to differences in exposure definitions, we repeated the analysis substituting 60-day and 30-day exposure windows for the 90-day exposure window. Third, to reduce the potential for confounding by indication for CCB and BB use, we directly compared current users with former users. Fourth, we repeated the analysis separating ischemic stroke into “unspecified stroke” and “specified ischemic stroke”. Analyses were performed using SAS version 9.2 (SAS Institute Inc., Cary, NC, USA). The study was approved by the Danish Data Protection Agency (record number 2011-41-5755). As this study did not involve contact with patients or any intervention, it was not required to obtain permission from the Danish Scientific Ethical Committee or to obtain patient consents.

## Results

### Patient characteristics

Patient characteristics are shown in Table 1. We identified 100,043 patients with a first-time stroke. Of these, 83,736 (83.7%) patients had ischemic stroke (median age: 74 years), 11,779 (11.8%) had ICH (median age: 72 years), and 4,528 (4.5%) had SAH (median age: 58 years). A total of 14,698 patients (14.7%) were current CCBs users, 3,446 (3.4%) were former users, and 81,899 (81.9%) were non-users. For BBs, the distribution was similar with a total of 19,515 (19.5%) current users, 3,609 (3.6%) former users, and 76,919 (76.9%) non-users. All cardiovascular diseases, obesity, diabetes mellitus, chronic kidney disease, and use of all cardiovascular drugs were more common among current users of CCB and BB in each stroke group than among non-users. Median age was also higher among current CCB/BB users than among non-users.

### Mortality

Overall 30-day mortality risk among non-users of CCB or BB was 10% for ischemic stroke, 33-34% for ICH, and 23% for SAH (Table 2). After multivariable adjustment, current use of CCBs or BBs was not associated with mortality compared with non-use for ischemic stroke [30-day MRR = 0.99 (95% confidence interval (CI): 0.94-1.05) for CCB and 1.01 (95% CI: 0.96-1.07) for BB], for ICH [30-day MRR = 1.05 (95% CI: 0.95-1.16) for CCB and 0.95 (95% CI: 0.87-1.04) for BB], or for SAH [30-day MRR = 1.05 (95% CI: 0.85-1.29) for CCBs and 0.89 (95% CI: 0.72-1.11) for BBs]. No association with mortality was found for former use of CCBs or BBs compared with non-use (Table 2).

**Table 2:** **Preadmission use of calcium channel blockers or beta blockers and 30-day mortality estimates following stroke**

	**Calcium channel blocker**			**Beta blocker**		
	**Mortality risk, % (95% CI)**	**Mortality rate ratio (95% CI)**		**Mortality risk, % (95% CI)**	**Mortality rate ratio (95% CI)**	
		**Unadjusted**	**Adjusted** ^**a**^		**Unadjusted**	**Adjusted** ^**a**^
**Ischemic stroke**						
Non-use	10.3 (10.1-10.5)	1 (reference)	1 (reference)	9.8 (9.5-10.0)	1 (reference)	1 (reference)
Former use	13.1 (11.9-14.3)	1.29 (1.16-1.43)	1.04 (0.94-1.15)	13.0 (11.8-14.2)	1.35 (1.22-1.49)	1.01 (0.91-1.12)
Current use	12.4 (11.9-13.0)	1.22 (1.15-1.29)	0.99 (0.94-1.05)	13.9 (13.3-14.4)	1.45 (1.38-1.52)	1.01 (0.96-1.07)
New use	10.1 (9.1-11.1)	0.97 (0.88-1.08)	0.89 (0.80-0.99)	12.6 (11.6-13.6)	1.31 (1.19-1.43)	0.97 (0.88-1.06)
Long-term use	13.3 (12.6-14.0)	1.31 (1.24-1.39)	1.03 (0.97-1.10)	14.3 (13.7-14.9)	1.50 (1.42-1.58)	1.02 (0.96-1.08)
**Intracerebral hemorrhage**						
Non-use	34.1 (33.2-35.0)	1 (reference)	1 (reference)	33.3 (32.4-34.3)	1 (reference)	1 (reference)
Former use	37.5 (32.6-42.9)	1.11 (0.93-1.32)	0.92 (0.77-1.10)	40.6 (35.8-45.8)	1.29 (1.09-1.52)	1.00 (0.84-1.19)
Current use	41.3 (38.6-44.2)	1.28 (1.16-1.41)	1.05 (0.95-1.16)	41.9 (39.7-44.2)	1.35 (1.25-1.46)	0.95 (0.87-1.04)
New use	36.6 (31.4-42.5)	1.09 (0.90-1.33)	0.95 (0.78-1.16)	41.7 (37.2-46.6)	1.36 (1.17-1.58)	0.96 (0.81-1.12)
Long-term use	42.9 (39.7-46.3)	1.35 (1.21-1.50)	1.09 (0.97-1.22)	42.0 (39.5-44.5)	1.35 (1.24-1.47)	0.94 (0.85-1.04)
**Subarachnoid hemorrhage**						
Non-use	22.6 (21.3-23.9)	1 (reference)	1 (reference)	22.8 (21.6-24.2)	1 (reference)	1 (reference)
Former use	32.2 (24.4-41.7)	1.51 (1.08-2.11)	1.05 (0.75-1.48)	25.0 (17.4-35.2)	1.12 (0.74-1.69)	0.60 (0.38-0.95)
Current use	33.0 (28.4-38.0)	1.56 (1.29-1.89)	1.05 (0.85-1.29)	31.6 (27.3-36.3)	1.46 (1.21-1.75)	0.89 (0.72-1.11)
New use	29.1 (21.0-39.5)	1.38 (0.94-2.02)	0.98 (0.66-1.45)	28.3 (20.5-38.3)	1.24 (0.85-1.81)	0.70 (0.46-1.06)
Long-term use	34.3 (28.9-40.2)	1.62 (1.31-2.01)	1.06 (0.84-1.34)	32.6 (27.7-38.1)	1.53 (1.25-1.88)	0.92 (0.72-1.17)

^a^Adjusted for sex, age groups, and the individual comorbidities and comedications listed in Table 1.

We found no substantial modification of the CCB or BB association (Additional file 1: Table S7). However, among ICH patients we found a reduction in MRR in younger users of both CCB (age <60 years) and BB (age between 60–69 years) although CIs were partly overlapping between age groups.

The results were robust in the analysis restricted to CT- or MRI-confirmed diagnoses (Additional file 1: Table S2) and in analyses using a 60-day (Additional file 1: Table S3) and 30-day (Additional file 1: Table S4) exposure window. Also, when current users were compared directly with former users (Additional file 1: Table S5) and when “unspecified stroke” and “specified ischemic stroke” were analyzed separately (Additional file 1: Table S6), no association with mortality following ischemic stroke, ICH, or SAH was found.

## Discussion

In this cohort study with virtually complete follow-up of 100,043 patients hospitalized for stroke, preadmission use of CCBs or BBs was not associated with 30-day mortality following ischemic stroke, ICH, or SAH. The results may be generalized to most industrial Western societies where changes in risk factor modification and treatment have followed international recommendations.

A number of studies have examined use of CCBs or BBs *after* stroke onset. A meta-analysis of 34 randomized controlled trials encompassing a total of 7,731 patients evaluated CCB administration *versus* control (placebo or standard medical treatment alone) after ischemic stroke onset and reported no beneficial effect on mortality (risk ratio = 1.07, 95% CI: 0.98-1.17) [20]. CCBs are prescribed as a component of vasospasm management after SAH onset and another recent meta-analysis reports more than 50% mortality reduction in SAH patients receiving this treatment (OR = 0.45, 95% CI: 0.15-1.29) [21]. As we studied the prognostic effect of CCB or BB use initiated before and not after hospitalization for stroke, our neutral results do not necessarily contradict these findings.

The effect of BB use *before* ischemic stroke on subsequent stroke severity has been examined in two cohort studies of 111 [22] and 1,375 [23] patients. Both studies reported a beneficial effect on stroke severity as measured by the Canadian Neurologic Scale, the National Institute of Health Stroke Scale [24], or the European Stroke Scale [25]. Although both studies examined pre-stroke use of BB, neither included mortality as an outcome measure. Consequently our results are not directly comparable.

It has recently been established that preadmission use of angiotensin-converting enzyme inhibitors and angiotensin receptor blockers reduces the 30-day mortality following ischemic stroke, whereas there was no association for ICH and SAH [12]. Our null results for ischemic stroke suggest that the positive effect of angiotensin-converting enzyme inhibitors and angiotensin receptor blockers on ischemic stroke mortality is mediated via pathways other than blood pressure control.

Transient hypertension is a common finding in the acute phase of stroke, and its management has been subject of debate [26]. A recent randomized trial of 4,071 patients, which examined the effect of blood pressure reduction in the acute phase of ischemic stroke, reported no difference between treatment groups on mortality up to 14 days or on major disability (OR = 1.00, 95% CI: 0.88-1.14) [27]. Our study supports a neutral effect of antihypertensive drug use on mortality following stroke, although we examined CBB and BB use initiated prior to stroke onset.

The rise in blood pressure during acute stroke was investigated in a recent population-based study of 653 patients with acute ischemic stroke or ICH [10]. Systolic blood pressure was substantially elevated compared with usual pre-stroke levels after intracerebral hemorrhage, whereas acute-phase systolic blood pressure after major ischemic stroke was only marginally raised. This suggests that the benefits of lowering blood pressure acutely after stroke might be expected to differ, favoring ICH [10]. Consistent with this interpretation, our results do not exclude a protective effect of preadmission use of CCB or BB among patients experiencing ICH at younger ages, although CIs were overlapping between strata of age (Additional file 1: Table S7).

A major strength of our study was its large size, providing statistically precise estimates. The study’s population-based design in the setting of a tax-supported universal healthcare system with unfettered access and complete follow-up of all patients largely eliminated selection bias. As well, the complete and prospectively recorded hospital and prescription histories reduced the risk of information biases [28].

A potential limitation is that our stroke data were derived from routine hospital discharge diagnoses, which may introduce coding errors [17]. The positive predictive value of acute stroke diagnoses in the DNRP has previously been validated and found to be approximately 97% for ischemic stroke, 74% for ICH, and 67% for SAH [17]. Misclassification due to coding errors would most likely be non-differential and thus could explain at least part of the neutral results in this study. However, in sensitivity analyses restricted to CT or MRI-confirmed cases, the results remained robust.

Because we classified *unspecified strokes* as ischemic strokes, a few ICHs (approximately 6%) were inevitably misclassified as ischemic strokes [17]. As we found no overall association between CBB or BB use and ICH mortality, such misclassification would bias the results for ischemic stroke towards the null.

Data on reimbursed medications are virtually complete in the prescription database [13], and neither CCB nor BB are sold over-the-counter in Denmark. We therefore identified all patients with redeemed prescriptions for CBB and BB. Still, use of redeemed prescriptions as a proxy for actual drug use may not always be accurate. We did base drug exposure information on actual dispensing at pharmacies rather than just on written prescriptions [13]. Mortality data, as recorded in the CRS, are virtually complete [14].

Although we controlled for several potential confounding factors, we cannot exclude unmeasured confounding. Moreover, we lacked information on smoking and blood pressure levels. However, we did adjust for hospital diagnoses of COPD and hypertension as proxy measures. Smoking and hypertension may worsen prognosis after stroke and are likely more prevalent among CCB and BB users than among non-users. Thus, such potential confounding would have biased our estimates towards increased mortality among CCB and BB users.

Although we adjusted for diagnoses of obesity, hospital-based registration is likely to be incomplete and residual confounding may persist. We lacked accurate information on Body Mass Index, which is a major limitation for adequately adjusting for obesity. However, we expect potential residual confounding to have negligible impact on the results.

## Conclusions

Preadmission use of CCBs or BBs was not associated with overall 30-day mortality among patients with ischemic stroke, ICH, or SAH.

## Additional file

Additional file 1:
**Supplemental online tables.**
