# Supplementary material for: Impact of preadmission treatment with calcium channel blockers or beta blockers on short-term mortality after stroke: a nationwide cohort study
Source: BMC Neurol. 2015 Mar 7;15:24. doi: 10.1186/s12883-015-0279-3 (PMC4365558; doi:10.1186/s12883-015-0279-3)
Supplement: Additional file 1: — Supplemental online tables. [file 12883_2015_279_MOESM1_ESM.pdf]

ONLINE SUPPLEMENT

Impact of Preadmission Treatment with Calcium Channel Blockers or Beta Blockers on Short-term Mortality after Stroke: A nationwide cohort study

Table S1. ICD and ATC codes.

**Stroke**	
Ischemic stroke	ICD-8: 433-434; ICD-10: I63-64
Intracerebral hemorrhage	ICD-8: 431; ICD-10: I61
Subarachnoid hemorrhage	ICD-8: 430; ICD-10: I60
CT scan	ICD-10: UXCA
MRI scan	ICD-10: UXMA
**Calcium channel blockers and beta blockers**	
Calcium channel blockers	ATC: C08
Beta blockers	ATC: C07
**Comedications**	
Cardiovascular drugs	
ACE-Is	ATC: C09A, C09B
ARBs	ATC: C09C, C09D
Diuretics	ATC: C03
Nitrates	ATC: C01DA
Statins	ATC: C10AA, C10B, B04AB
Aspirin	ATC: B01AC06, N02BA01
Clopidogrel	ATC: B01AC04
Vitamin K antagonists	ATC: B01AA03, B01AA04
Other drugs	
Systemic glucocorticoids	ATC: H02AB
SSRIs	ATC: N06AB
Bisphosphonates	ATC: M05BA-B
NSAIDs	ATC: M01A
**Individual comorbidities**	
Cardiovascular diseases	
Congestive heart failure	ICD-8: 427.09, 427.10, 427.11, 427.19, 428.99, 782.49; ICD-10: I50, I11.0, I13.0, I13.2
Myocardial infarction	ICD-8: 410; ICD-10: I21, I22, I23
Angina pectoris	ICD-8: 413; ICD-10: I20.9, I25.1, I25.9
Atrial fibrillation or flutter	ICD-8: 427.93, 427.94; ICD-10: I48
Heart valve disease	ICD-8: 394-398; ICD-10: I05, I06, I07, I08.0, I09.8, I34-I37, I39.0, I39.3, I51.1A, Q22
Intermittent arterial claudication	ICD-8: 443.89-443.99; ICD-10: I73.9
Venous thromboembolism	ICD-8: 451.00; ICD-10: I80.1-3, I26
Hypertension	ICD-8: 400-404; ICD-10: I10-I15
Other diseases	
Obesity	ICD-8: 277; ICD-10: E65-E68
Diabetes	ICD-8: 249-250; ICD-10: E10-E14, O24 (except O24.4), H36.0; ATC: A10B, A10A
Chronic kidney disease	ICD-8: 249.02, 250.02, 753.10-753.19, 582, 583, 584, 590.09, 593.20, 792; ICD-10: E10.2, E11.2, E14.2, N03, N05, N11.0, N14, N16, N18-N19, N26.9, Q61.1-Q61.4
COPD	ICD-8: 491-492; ICD-10: J41-44; ATC: R03
Alcoholism-related diseases	ICD-8: 291, 303, 456, 571.09, 571.10, 577.10; ICD-10: F10.1-9, G31.2, G62.1, G72.1, I 42.6, K29.2, K86.0, Z72.1; ATC: N07BB
Cancer	ICD-8: 140-207; ICD-10: C00-C96
Dementia	ICD-8: 290.09-290.19, 293.09; ICD-10: F00-F03, F05.1, G30

ACE-Is indicates angiotensin-converting enzyme inhibitors; ARBs, angiotensin receptor blockers; SSRIs, selective serotonin reuptake inhibitors; NSAIDs, nonsteroidal anti-inflammatory drugs; COPD, chronic obstructive pulmonary disease.

Table S2. Sensitivity analysis: Preadmission calcium channel blocker or beta blocker use and 30-day mortality estimates following stroke, with confirmation of diagnosis with CT or MRI scan.

	Calcium channel blocker			Beta blocker		
	**Mortality risk, % (95% CI)**	**Mortality rate ratio (95% CI)**		**Mortality risk, % (95% CI)**	**Mortality rate ratio (95% CI)**	
		Unadjusted	Adjusted[a]		Unadjusted	Adjusted[a]
**Ischemic stroke**						
Non-use	9.2 (9.0-9.5)	1 (reference)	1 (reference)	8.7 (8.4-8.9)	1 (reference)	1 (reference)
Former use	12.0 (10.8-13.4)	1.32 (1.18-1.48)	1.03 (0.92-1.16)	12.6 (11.4-13.9)	1.48 (1.33-1.66)	1.07 (0.95-1.20)
Current use	11.5 (10.9-12.1)	1.26 (1.19-1.34)	1.00 (0.94-1.06)	12.9 (12.4-13.5)	1.53 (1.45-1.61)	1.03 (0.97-1.09)
New use	9.2 (8.3-10.3)	1.00 (0.89-1.13)	0.89 (0.79-1.00)	11.7 (10.7-12.8)	1.37 (1.24-1.51)	0.97 (0.87-1.08)
Long-term use	12.4 (11.7-13.1)	1.36 (1.27-1.45)	1.04 (0.97-1.11)	13.3 (12.7-14.0)	1.58 (1.49-1.67)	1.04 (0.98-1.11)
**Intracerebral hemorrhage**						
Non-use	34.1 (33.1-35.1)	1 (reference)	1 (reference)	33.3 (32.3-34.3)	1 (reference)	1 (reference)
Former use	37.7 (32.5-43.4)	1.11 (0.92-1.34)	0.93 (0.77-1.12)	40.2 (35.2-45.6)	1.27 (1.07-1.51)	0.99 (0.83-1.19)
Current use	42.1 (39.2-45.2)	1.32 (1.19-1.46)	1.08 (0.97-1.20)	42.4 (40.1-44.7)	1.37 (1.27-1.49)	0.97 (0.88-1.07)
New use	38.5 (32.9-44.7)	1.18 (0.96-1.44)	1.02 (0.83-1.25)	43.7 (38.9-48.9)	1.45 (1.24-1.69)	1.04 (0.88-1.22)
Long-term use	43.3 (39.9-46.8)	1.37 (1.22-1.53)	1.10 (0.98-1.24)	42.2 (39.5-44.7)	1.35 (1.24-1.48)	0.95 (0.85-1.05)
**Subarachnoid hemorrhage**						
Non-use	23.1 (21.8-24.5)	1 (reference)	1 (reference)	23.2 (21.9-24.6)	1 (reference)	1 (reference)
Former use	34.1 (25.5-44.6)	1.59 (1.12-2.26)	1.10 (0.76-1.59)	29.1 (20.4-40.5)	1.31 (0.87-1.98)	0.66 (0.41-1.04)
Current use	34.3 (29.5-39.7)	1.61 (1.32-1.95)	1.07 (0.86-1.32)	33.6 (29.0-38.7)	1.54 (1.28-1.86)	0.92 (0.73-1.15)
New use	29.8 (21.2-40.8)	1.40 (0.94-2.08)	0.95 (0.63-1.43)	28.2 (20.2-38.7)	1.22 (0.82-1.80)	0.65 (0.42-1.00)
Long-term use	35.8 (30.2-42.1)	1.68 (1.35-2.09)	1.10 (0.87-1.39)	35.4 (30.1-41.3)	1.66 (1.35-2.04)	0.98 (0.76-1.26)

[a]Adjusted for sex, age groups, and individual comorbidities and comedications listed in Table 1.

Table S3. Sensitivity analysis: Preadmission calcium channel blocker or beta blocker use and 30-day mortality estimates following stroke, with 60-day exposure window.

	Calcium channel blocker			Beta blocker		
	**Mortality risk, % (95% CI)**	**Mortality rate ratio (95% CI)**		**Mortality risk, % (95% CI)**	**Mortality rate ratio (95% CI)**	
		Unadjusted	Adjusted[a]		Unadjusted	Adjusted[a]
**Ischemic stroke**						
Non-use	10.3 (10.1-10.5)	1 (reference)	1 (reference)	9.8 (9.5-10.0)	1 (reference)	1 (reference)
Former use	12.6 (11.8-13.4)	1.24 (1.15-1.33)	1.02 (0.95-1.10)	13.0 (12.2-13.8)	1.36 (1.26-1.46)	1.01 (0.93-1.09)
Current use	12.5 (11.9-13.2)	1.23 (1.16-1.30)	0.99 (0.93-1.06)	14.1 (13.5-14.6)	1.47 (1.40-1.55)	1.01 (0.95-1.06)
New use	8.6 (7.4-9.9)	0.82 (0.70-0.96)	0.80 (0.68-0.94)	13.4 (12.1-14.9)	1.40 (1.25-1.58)	1.07 (0.95-1.21)
Long-term use	13.4 (12.7-14.2)	1.32 (1.24-1.41)	1.03 (0.96-1.10)	14.2 (13.5-14.8)	1.48 (1.41-1.57)	1.00 (0.94-1.06)
**Intracerebral hemorrhage**						
Non-use	34.1 (33.2-35.0)	1 (reference)	1 (reference)	33.3 (32.4-34.3)	1 (reference)	1 (reference)
Former use	39.5 (35.8-43.4)	1.20 (1.05-1.36)	1.00 (0.87-1.14)	39.9 (36.6-43.5)	1.27 (1.12-1.42)	0.94 (0.83-1.07)
Current use	41.2 (38.0-44.5)	1.27 (1.14-1.42)	1.04 (0.93-1.16)	42.6 (40.1-45.1)	1.38 (1.27-1.50)	0.96 (0.87-1.06)
New use	34.9 (27.5-43.6)	1.00 (0.75-1.33)	0.88 (0.66-1.17)	42.9 (36.4-49.9)	1.42 (1.15-1.75)	0.97 (0.77-1.20)
Long-term use	42.4 (38.9-46.0)	1.33 (1.18-1.49)	1.07 (0.94-1.20)	42.5 (39.9-45.3)	1.37 (1.26-1.50)	0.95 (0.86-1.05)
**Subarachnoid hemorrhage**						
Non-use	22.6 (21.3-23.9)	1 (reference)	1 (reference)	22.8 (21.6-24.2)	1 (reference)	1 (reference)
Former use	31.5 (25.6-38.3)	1.48 (1.15-1.90)	1.02 (0.78-1.32)	23.9 (18.5-30.7)	1.07 (0.79-1.44)	0.61 (0.44-0.85)
Current use	33.8 (28.5-39.8)	1.60 (1.29-1.99)	1.06 (0.84-1.34)	34.2 (29.2-39.7)	1.60 (1.31-1.95)	0.96 (0.76-1.22)
New use	23.9 (14.0-39.0)	1.10 (0.61-1.99)	0.79 (0.43-1.45)	34.5 (23.8-48.2)	1.56 (1.00-2.42)	0.84 (0.52-1.35)
Long-term use	35.8 (29.9-42.5)	1.71 (1.36-2.15)	1.11 (0.86-1.41)	34.1 (28.7-40.3)	1.61 (1.29-2.00)	0.97 (0.75-1.25)

[a]Adjusted for sex, age groups, and individual comorbidities and comedications listed in Table 1.

Table S4. Sensitivity analysis: Preadmission calcium channel blocker or beta blocker use and 30-day mortality estimates following stroke, with 30-day exposure window.

	Calcium channel blocker			Beta blocker		
	Mortality risk, % (95% CI)	Mortality rate ratio (95% CI)		Mortality risk, % (95% CI)	Mortality rate ratio (95% CI)	
		Unadjusted	Adjusted[a]		Unadjusted	Adjusted[a]
**Ischemic stroke**						
Non-use	10.3 (10.1-10.5)	1 (reference)	1 (reference)	9.8 (9.5-10.0)	1 (reference)	1 (reference)
Former use	12.4 (11.8-13.1)	1.22 (1.15-1.29)	1.02 (0.95-1.08)	12.8 (12.2-13.4)	1.33 (1.26-1.41)	0.99 (0.93-1.05)
Current use	12.8 (11.9-13.6)	1.25 (1.16-1.35)	0.98 (0.91-1.06)	15.0 (14.2-15.8)	1.58 (1.48-1.68)	1.03 (0.96-1.10)
New use	8.3 (6.7-10.2)	0.79 (0.63-0.99)	0.79 (0.63-0.98)	12.4 (10.6-14.5)	1.29 (1.09-1.52)	1.01 (0.86-1.20)
Long-term use	13.6 (12.7-14.6)	1.34 (1.24-1.45)	1.01 (0.93-1.10)	15.4 (14.6-16.2)	1.62 (1.52-1.73)	1.04 (0.97-1.11)
**Intracerebral hemorrhage**						
Non-use	34.1 (33.2-35.0)	1 (reference)	1 (reference)	33.3 (32.4-34.3)	1 (reference)	1 (reference)
Former use	40.1 (37.1-43.2)	1.22 (1.10-1.36)	1.01 (0.91-1.13)	40.7 (38.2-43.3)	1.30 (1.19-1.42)	0.96 (0.87-1.06)
Current use	41.2 (37.1-45.5)	1.28 (1.11-1.47)	1.03 (0.90-1.19)	43.3 (40.1-46.6)	1.40 (1.26-1.56)	0.93 (0.83-1.05)
New use	31.1 (21.9-43.0)	0.88 (0.58-1.32)	0.80 (0.53-1.20)	40.2 (31.0-51.0)	1.29 (0.93-1.78)	0.88 (0.63-1.22)
Long-term use	42.9 (38.4-47.6)	1.35 (1.17-1.56)	1.07 (0.92-1.25)	43.6 (40.3-47.2)	1.42 (1.27-1.58)	0.93 (0.83-1.06)
**Subarachnoid hemorrhage**						
Non-use	22.6 (21.3-23.9)	1 (reference)	1 (reference)	22.8 (21.6-24.2)	1 (reference)	1 (reference)
Former use	33.6 (28.8-38.9)	1.59 (1.31-1.93)	1.07 (0.87-1.32)	27.6 (23.1-32.8)	1.25 (1.01-1.56)	0.75 (0.59-0.97)
Current use	30.7 (23.8-39.1)	1.45 (1.07-1.96)	0.97 (0.71-1.34)	35.4 (28.9-42.9)	1.66 (1.28-2.14)	0.95 (0.70-1.27)
New use	21.1 (8.5-46.8)	0.97 (0.36-2.59)	0.97 (0.36-2.63)	31.8 (16.6-55.4)	1.39 (0.66-2.92)	0.77 (0.35-1.69)
Long-term use	32.3 (24.7-41.4)	1.52 (1.11-2.10)	0.97 (0.69-1.36)	35.9 (28.9-44.0)	1.70 (1.30-2.23)	0.96 (0.71-1.31)

[a]Adjusted for sex, age groups, and individual comorbidities and comedications listed in Table 1.

Table S5. Sensitivity analysis: Current vs. former calcium channel blocker or beta blocker use and 30-day mortality estimates following stroke.

	Calcium channel blocker		Beta blocker	
	Mortality rate ratio (95% CI)		Mortality rate ratio (95% CI)	
	Unadjusted	Adjusted[a]	Unadjusted	Adjusted[a]
Ischemic stroke (current users)	0.94 (0.85-1.06)	0.94 (0.84-1.05)	1.07 (0.97-1.19)	1.00 (0.90-1.11)
Intracerebral hemorrhage (current users)	1.16 (0.95-1.41)	1.20 (0.98-1.47)	1.05 (0.88-1.25)	0.99 (0.83-1.19)
Subarachnoid hemorrhage (current users)	1.03 (0.71-1.50)	0.97 (0.66-1.43)	1.30 (0.84-2.03)	1.39 (0.86-2.23)

[a]Adjusted for sex, age groups, and individual comorbidities and comedications listed in Table 1.

Table S6. Sensitivity analysis: Preadmission use of calcium channel blockers or beta blockers and 30-day mortality estimates following ischemic stroke (combined, specified, and unspecified).

	Calcium channel blocker			Beta blocker		
	Mortality risk, % (95% CI)	Mortality rate ratio (95% CI)		Mortality risk, % (95% CI)	Mortality rate ratio (95% CI)	
		Unadjusted	Adjusted[a]		Unadjusted	Adjusted[a]
**Combined ischemic stroke (I63 + I64)**						
Nonuse	10.3 (10.1-10.5)	1 (reference)	1 (reference)	9.8 (9.5-10.0)	1 (reference)	1 (reference)
Former use	13.1 (11.9-14.3)	1.29 (1.16-1.43)	1.04 (0.94-1.15)	13.0 (11.8-14.2)	1.35 (1.22-1.49)	1.01 (0.91-1.12)
Current use	12.4 (11.9-13.0)	1.22 (1.15-1.29)	0.99 (0.94-1.05)	13.9 (13.3-14.4)	1.45 (1.38-1.52)	1.01 (0.96-1.07)
New use	10.1 (9.1-11.1)	0.97 (0.88-1.08)	0.89 (0.80-0.99)	12.6 (11.6-13.6)	1.31 (1.19-1.43)	0.97 (0.88-1.06)
Long-term use	13.3 (12.6-14.0)	1.31 (1.24-1.39)	1.03 (0.97-1.10)	14.3 (13.7-14.9)	1.50 (1.42-1.58)	1.02 (0.96-1.08)
**Specified ischemic stroke (I64)**						
Nonuse	8.1 (7.85-8.37)	1 (reference)	1 (reference)	7.5 (7.3-7.8)	1 (reference)	1 (reference)
Former use	10.7 (9.39-12.24)	1.35 (1.17-1.56)	1.09 (0.94-1.26)	10.8 (9.4-12.2)	1.45 (1.26-1.67)	1.01 (0.87-1.17)
Current use	10.3 (9.66-11.00)	1.29 (1.19-1.39)	1.03 (0.95-1.11)	11.9 (11.3-12.6)	1.62 (1.52-1.73)	1.04 (0.97-1.13)
New use	8.5 (7.40-9.73)	1.05 (0.91-1.22)	0.95 (0.82-1.10)	10.4 (9.3-11.7)	1.41 (1.24-1.60)	0.96 (0.84-1.10)
Long-term use	11.0 (10.22-11.84)	1.38 (1.27-1.50)	1.05 (0.96-1.15)	12.4 (11.7-13.1)	1.69 (1.57-1.82)	1.06 (0.98-1.16)
**Unspecified stroke (I64)**						
Nonuse	13.8 (13.35-14.19)	1 (reference)	1 (reference)	13.3 (12.9-13.8)	1 (reference)	1 (reference)
Former use	16.7 (14.72-19.00)	1.23 (1.06-1.42)	1.00 (0.87-1.16)	16.5 (14.5-18.7)	1.26 (1.09-1.45)	1.03 (0.89-1.20)
Current use	15.6 (14.68-16.66)	1.15 (1.06-1.24)	0.97 (0.89-1.05)	16.8 (15.9-17.7)	1.29 (1.20-1.38)	0.99 (0.92-1.07)
New use	12.6 (10.92-14.43)	0.91 (0.78-1.05)	0.84 (0.72-0.99)	15.9 (14.2-17.7)	1.20 (1.06-1.37)	0.99 (0.87-1.13)
Long-term use	16.8 (15.60-17.98)	1.24 (1.14-1.35)	1.02 (0.93-1.11)	17.1 (16.1-18.2)	1.31 (1.22-1.41)	0.99 (0.91-1.08)

[a]Adjusted for sex, age groups, and individual comorbidities and comedications listed in Table 1.

Table S7. Thirty-day mortality rate ratios following ischemic stroke, intracerebral hemorrhage, and subarachnoid hemorrhage, by sex, age, and comorbidity among current vs. non-users of calcium channel blockers or beta blockers.

	Calcium channel blocker			Beta blocker		
	Mortality rate ratios (95% CI)[a]			Mortality rate ratios (95% CI)[a]		
	Ischemic stroke	ICH	SAH	Ischemic stroke	ICH	SAH
**Sex**						
Male	0.97 (0.88-1.06)	0.97 (0.84-1.12)	1.02 (0.74-1.40)	1.01 (0.92-1.09)	0.93 (0.81-1.06)	0.81 (0.58-1.14)
Female	1.00 (0.93-1.08)	1.13 (0.99-1.30)	1.03 (0.78-1.36)	1.01 (0.94-1.08)	0.98 (0.86-1.11)	0.97 (0.73-1.30)
**Age**						
<60	0.80 (0.57-1.12)	0.65 (0.43-0.97)	1.00 (0.59-1.71)	1.02 (0.76-1.37)	1.26 (0.92-1.71)	1.00 (0.60-1.67)
60-69	0.83 (0.69-1.00)	1.03 (0.80-1.33)	0.97 (0.59-1.57)	1.06 (0.89-1.25)	0.71 (0.56-0.91)	0.90 (0.57-1.44)
70-79	0.99 (0.88-1.11)	0.98 (0.81-1.17)	1.13 (0.74-1.70)	0.98 (0.88-1.10)	1.06 (0.90-1.24)	0.74 (0.48-1.16)
≥80	1.03 (0.96-1.10)	1.19 (1.02-1.38)	0.98 (0.69-1.39)	1.01 (0.95-1.08)	0.90 (0.78-1.03)	0.94 (0.64-1.39)
**Cardiovascular diseases**						
Myocardial infarction	0.80 (0.68-0.93)	1.01 (0.76-1.34)	1.08 (0.39-2.93)	0.88 (0.77-1.00)	0.77 (0.59-1.00)	1.13 (0.53-2.41)
No myocardial infarction	1.02 (0.96-1.09)	1.05 (0.94-1.17)	1.06 (0.85-1.31)	1.04 (0.98-1.10)	0.98 (0.89-1.08)	0.91 (0.72-1.15)
Congestive heart failure	0.87 (0.76-0.99)	0.74 (0.55-1.01)	1.90 (0.71-5.10)	0.87 (0.78-0.97)	0.85 (0.67-1.08)	0.51 (0.24-1.07)
No congestive heart failure	1.02 (0.95-1.08)	1.10 (0.99-1.22)	1.03 (0.83-1.28)	1.05 (0.98-1.11)	0.97 (0.87-1.07)	0.95 (0.76-1.20)
Atrial fibrillation or flutter	0.97 (0.87-1.07)	0.94 (0.77-1.14)	1.00 (0.58-1.72)	0.99 (0.90-1.08)	0.93 (0.79-1.09)	0.89 (0.51-1.55)
No atrial fibrillation or flutter	1.00 (0.93-1.07)	1.08 (0.96-1.22)	1.02 (0.81-1.28)	1.01 (0.94-1.08)	0.95 (0.85-1.06)	0.87 (0.68-1.12)
Angina pectoris	0.94 (0.84-1.05)	1.10 (0.90-1.35)	0.99 (0.59-1.64)	0.97 (0.87-1.07)	0.86 (0.72-1.04)	0.67 (0.41-1.08)
No angina pectoris	1.01 (0.94-1.08)	1.01 (0.90-1.14)	1.05 (0.83-1.33)	1.02 (0.96-1.08)	0.98 (0.88-1.09)	1.02 (0.80-1.30)
Hypertension	1.01 (0.93-1.09)	1.06 (0.92-1.22)	0.81 (0.59-1.11)	0.96 (0.88-1.04)	0.95 (0.83-1.09)	0.82 (0.59-1.14)
No hypertension	0.97 (0.90-1.05)	1.03 (0.89-1.19)	1.15 (0.87-1.53)	1.04 (0.97-1.11)	0.95 (0.84-1.07)	0.90 (0.67-1.21)
**Other diseases**						
Diabetes	1.02 (0.91-1.16)	1.03 (0.81-1.32)	1.16 (0.61-2.24)	1.05 (0.93-1.18)	0.91 (0.73-1.15)	0.84 (0.39-1.82)
No diabetes	0.98 (0.92-1.04)	1.04 (0.93-1.17)	1.03 (0.82-1.29)	1.00 (0.94-1.06)	0.96 (0.87-1.06)	0.91 (0.72-1.15)
Kidney disease	1.01 (0.81-1.27)	1.37 (0.94-1.99)	-	0.88 (0.71-1.09)	0.95 (0.66-1.37)	1.06 (0.16-6.86)
No kidney disease	0.99 (0.93-1.05)	1.02 (0.92-1.14)	1.01 (0.81-1.25)	1.02 (0.96-1.08)	0.95 (0.86-1.04)	0.87 (0.69-1.09)

[a]Adjusted for sex, age groups, and individual comorbidities and comedications listed in Table 1.
